# Nanomaterials for Persistent Organic Pollutants Decontamination in Water: Mechanisms, Challenges, and Future Perspectives

**DOI:** 10.3390/nano15141133

**Published:** 2025-07-21

**Authors:** Risky Ayu Kristanti, Tony Hadibarata, Adelina-Gabriela Niculescu, Dan Eduard Mihaiescu, Alexandru Mihai Grumezescu

**Affiliations:** 1 Research Center for Oceanography, National Research and Innovation Agency, Pasir Putih I, Jakarta 14430, Indonesia; risky.ayu.kristanti@brin.go.id; 2 Department of Science and Engineering of Oxide Materials and Nanomaterials, National University of Science and Technology Politehnica Bucharest, 011061 Bucharest, Romania; adelina.niculescu@upb.ro (A.-G.N.); dan.mihaiescu@upb.ro (D.E.M.); agrumezescu@upb.ro (A.M.G.); 3 Environmental Engineering Program, Faculty of Engineering and Science, Curtin University Malaysia, CDT 250, Miri 98009, Malaysia; 4 Research Institute of the University of Bucharest—ICUB, University of Bucharest, 050657 Bucharest, Romania

**Keywords:** persistent organic pollutants, POPs, nanomaterials, water decontamination, toxicity assessment, environmental fate, scalability challenges

## Abstract

Nanomaterials possess unique physicochemical properties that position them as promising candidates for environmental remediation, particularly in the removal of persistent organic pollutants (POPs) from aqueous systems. Their high surface area, tunable functionality, and strong adsorption capabilities have attracted significant attention. In this context, this paper reviews the mechanisms of nanomaterial-based POP decontamination, also providing a critical overview of the limitations and challenges in applying these methods. Specifically, issues of stability, reusability, and aggregation are discussed, which can lead to performance decay during repeated use. In addition, the practical application requires nanocomposites to enable efficient separation and mitigate agglomeration. Environmental concerns also arise from nanomaterials’ fate, transport, and potential toxicity, which may impact aquatic ecosystems and non-target organisms. When checking for large-scale application feasibility, impurities typically add to production costs, recovery problems, and general infrastructure limitations. In addition to these points, there are no standard guidelines or clear risk assessment procedures for registering a product. Unprecedented cross-disciplinary research between natural, human, and technological studies and outreach programs is needed to facilitate the development and diffusion of the results. The barriers will eventually be breached to move from laboratory success in developing the desperately needed new water purification technologies to field-ready water treatment solutions that can address the global POP contamination problem.

## 1. Introduction

Persistent organic pollutants (POPs) are toxic chemical substances that persist in the environment, where they bioaccumulate in the food chain and pose potential threats to humans and the general ecology. They exist in the form of a wide variety of compounds, including pesticides (DDT or aldrin), industrial chemicals (polychlorinated biphenyls or PCBs), and unintentionally formed chemicals (dioxins and furans). In its requirement for protecting human health and the environment, particularly for the fragile ecosystem, the Stockholm Convention, an international environmental treaty, classifies POPs into several categories: intentionally produced POPs, unintentionally produced POPs, and those subject to elimination or restriction [1,2,3].

The primary sources of POP contamination can be noted due to the runoff, point source industrial discharge, hazardous waste mismanagement, atmospheric deposition, and the burning of organic matter. These pollutants may enter aquatic ecosystems directly through the discharge of effluents and emissions into the air, followed by atmospheric deposition [4,5]. Once inside, since many POPs have an affinity for binding to sediments or staying colloidal in water, they eventually become a long-term risk of contamination. Mobilization of these pollutants may occur under specific conditions, such as pH variations and the redox potential, as well as disturbances in the sediment layers. Mobilization can lead to the recontamination of the water column and, subsequently, aquatic food chains where the organisms tend to once again take up these substances [6,7].

POPs are notorious for their high toxicity, even at low concentrations. They can cause a range of adverse effects in both humans and wildlife, including endocrine disruption, neurotoxicity, reproductive issues, immunosuppression, and carcinogenicity. One of the most significant concerns with POPs is their ability to bioaccumulate in living organisms. Lipophilic in nature, these compounds are stored in fatty tissues and biomagnify as they move up the food chain—from plankton to fish to apex predators, including humans. Prolonged exposure to POPs has been linked to developmental problems in children, liver damage, and increased susceptibility to diseases [1,3,5].

POPs can travel long distances from their original source through atmospheric and oceanic currents. This long-range transport contributes to global distribution, with traces of POPs detected even in remote regions such as the Arctic and Antarctic. Their environmental persistence, often measured in years or even decades, means they remain active in ecosystems for extended periods. This persistence complicates remediation efforts and leads to cumulative exposure risks over time. Additionally, their stability under natural conditions allows them to be transported intact over vast areas, exacerbating their ecological footprint [6,8].

Conventional water treatment methods such as coagulation, flocculation, sedimentation, and biological treatment are generally ineffective in removing POPs due to their hydrophobicity, low aqueous solubility, and chemical stability. These methods are designed to remove suspended solids, biodegradable organic matter, and certain pathogens, but do not target micro-pollutants like POPs. Biological treatments, in particular, fail to degrade POPs effectively because the compounds resist microbial breakdown due to their complex molecular structures [9,10,11].

Given the limitations of traditional methods, there is a pressing need for advanced treatment technologies capable of removing or degrading POPs from water systems. Techniques such as advanced oxidation processes (AOPs), membrane filtration, and adsorption using activated carbon have been explored with varying success. However, these methods often come with high operational costs, limited regeneration potential, or incomplete degradation, necessitating the exploration of more efficient and cost-effective alternatives such as nanomaterials [9,12,13].

Nanomaterials offer significant advantages in remediation because of their high surface area-to-volume ratio, tunable surface properties, and enhanced reactivity [14], especially in the remediation of POPs. The molecules of these materials can interact with POPs, enabling adsorption, degradation, or transformation processes [13]. For example, carbonaceous nanomaterials such as graphene oxide and carbon nanotubes have been reported to exhibit high hydrophobic organic pollutant sorption capacities. Zero-valent iron and titanium dioxide are among the metal and metal oxide nanoparticles known to catalyze the type of redox reaction responsible for the decomposition of POPs into less harmful compounds [10,15].

Recent advances in nanotechnology have resulted in the emergence of multifunctional mechanisms in nanocomposites combining adsorption, catalysis, and photodegradation processes [16]. The major approach has been an efficient visible light-activated photocatalyst convertible to the degradation of POPs without secondary pollution [12,17,18,19]. In addition, magnetic nanomaterials are easily recoverable using magnets to facilitate recycling [7], thereby reducing the overall treatment cost. There has been great interest in using synthesis methodologies for nanomaterials via plant extract or microbial pathways, which are environmentally benign methodologies in accordance with the direction for sustainability [20,21,22]. In this context, this review uniquely integrates the removal mechanisms, environmental impacts, and practical deployment strategies of nanomaterials in the remediation of POPs, bridging the gap between laboratory research results and practical applications. Different from previous reviews, this review focuses on emerging nanomaterials, green synthesis methods, and regulatory perspectives, aiming to comprehensively understand the role of nanotechnology in addressing the problem of POPs.

## 2. Properties and Characteristics of POPs Affecting Their Removal

### 2.1. Chemical and Physical Properties

POPs have characteristic chemical and physical properties, making them persistent in the environment and thus posing problems in water purification processes [4,23]. The hydrophobic and low water solubility of POPs are definitive for their contamination potential. POPs are not uniformly distributed in the water but are adsorbed on organic matter or deposited in sediments and biota; therefore, they are less amenable to direct chemical or biological attack [2,5,23]. In addition to hydrophobicity, POPs are highly resistant to chemical degradation. Most compounds contain halogenated aromatic structures, especially chlorinated rings, which make them resistant to hydrolytic, oxidative, or microbial cleavage. This chemical inactivity makes POPs stubborn under natural environmental conditions and during typical treatment processes. Their stability also allows long-term persistence in water systems, making a long exposure time possible and increasing ecological risk [10].

### 2.2. Environmental Fate and Transport

The environmental characteristics of POPs complicate their remediation. The lipophilic and stable nature of the POPs enhances their accumulation in aquatic organisms through biomagnification. The trophic transfer process ensures bioconcentration of the relatively small aquatic biota consumed by fish, birds, mammals, and humans. This causes a major concern in remediation strategies since most technologies being considered for treatment are mass- and volume-transfer-limited. The resuspension increases mass transfer rates, breaking through the equilibrium attained during sedimentation. A dietary food chain in which fish is at the highest possible position amplifies the health hazard associated with ecological toxicity through bioconcentration [24,25]. Strong binding also occurs between the POPs and either sediment particles or humic substances; hence, degradation is protected directly, but has the added disadvantage of being a long-term source of contamination. Some more works also propose that naturally existing or synthesized nanoparticles in aqueous systems can equally absorb POPs, changing their environmental transport and reactivity. Such interactions show the complicated matrix in which POPs exist and the need for remediation strategies [26,27].

### 2.3. Challenges in Removing POPs from Water

Inherent with the properties of POPs, several challenges are notable regarding their remediation from contaminated water. A major one is the mineralization of the compounds. Indeed, advanced oxidation processes or nanomaterial-based treatments will only partially convert POPs into less harmful end-products such as carbon dioxide and water. Normally, most compounds are very stable intermediates that transform to a stage where the toxicity or reactivity is lost [18]. Equally important is the fact that treatment may result in even more toxic products. For instance, the partial oxidation or photolysis of some POPs may form dioxin-like compounds that usually possess higher toxicity than the parent compounds. Moreover, these byproducts are mostly analytically problematic and can easily pass undetected, especially in complex water matrices; thus, careful monitoring is essential to ensure comprehensive treatments that can safely manage all the intermediates resulting from the decomposition of the POPs [10,28].

## 3. Nanomaterials Used for POPs Decontamination

**Figure 1:**
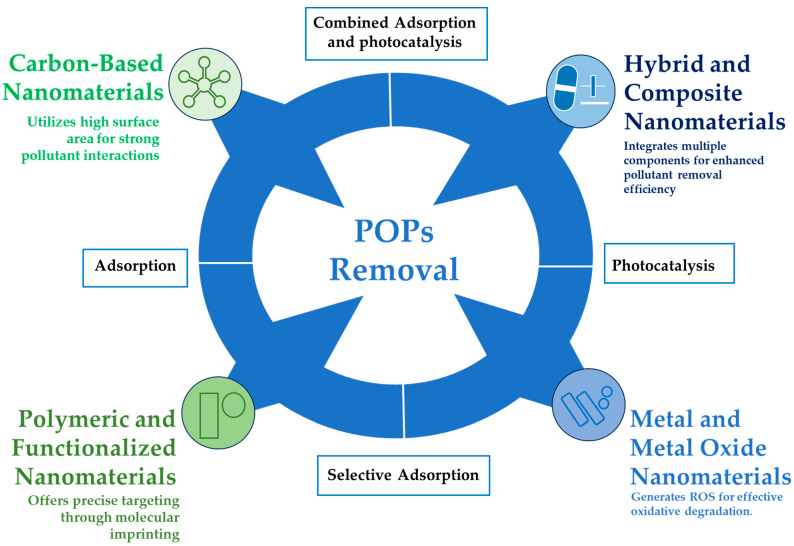
Nanomaterials used for the decontamination of persistent organic pollutants (POPs).

The use of nanomaterials has emerged as a promising solution for the decontamination of POPs in aquatic environments (Figure 1). Because of their high surface area-to-volume ratio, tunable surface properties, and ability to incorporate multiple functionalities, they are superior to conventional adsorbents and catalysts. Mostly, the action of different mechanisms is provided by nanomaterial-adsorption, photocatalysis, and reductive dechlorination, which in turn helps the degradation or elimination of very stable POPs [13,29,30].

### 3.1. Carbon-Based Nanomaterials

Two of the most studied carbon-based nanomaterials for removing POPs are graphene oxide and carbon nanotubes. These compounds have highly superior adsorption capacities due to their surface areas, and the porous structure allows π–π interactions with aromatic compounds. Oxygen-containing functional groups in graphene oxide make way for the modification of the material and, consequently, stronger interactions with halogenated POPs, such as PCBs and organochlorine pesticides [15]. Equally, multi-walled CNTs have been more effective for the sorption of POPs, mostly by hydrophobic interactions and van der Waals forces [26]. Activated carbons, in nanocomposite form, include all modernization of conventional activated carbons where the improvement can be at the nanoscale and/or the attachment of alternative elements like metal oxides or polymers [31]. These nanocomposites show higher efficiency and selectivity toward different POPs due to the combination of effects between the constituents of the nanomaterials. For example, a magnetite-modified activated carbon can be separated with simple magnetic processes after pollutants are adsorbed from the water, thus cleaning the water and making it more applicable [32].

### 3.2. Metal and Metal Oxide Nanoparticles

Metal and metal oxide nanoparticles exhibit catalytic properties that make them very suitable for attacking the stable molecular structures of POPs. Titanium dioxide and zinc oxide are active under UV or visible light as photocatalysts. When irradiated, these semiconductors will give hydroxyl radicals, and superoxide anions in active oxygen species can degrade the POPs in oxidative pathways [18,29,33]. A great deal of research has been conducted on photodegrading dioxins, PCBs, and polycyclic aromatic hydrocarbons by TiO_2_ to intermediate products of less toxicity or mineralized products with fewer toxic components [34]. Nano zero-valent iron (nZVI) has also emerged as a novel strong nanomaterial for the remediation of POPs in dechlorination and offers promising results in reducing higher chlorinated forms to lower ones through providing electrons with carbon-halogen bonds of pollutants to transform them into less chlorinated, more biodegradable forms. This has been effectively used to break down chlorinated chemicals and bug killers in groundwater and surface water bodies. Even though they are reactive, nano zero-valent iron particles often group together and turn into rust, so surface changes or mixing with stabilizers is performed to make them work better [35].

### 3.3. Polymeric and Functionalized Nanomaterials

Molecularly imprinted polymers (MIPs) are synthetic nanomaterials providing specific binding sites designed specifically for the target POPs. Binding sites are actually synthesized during the process of polymerization by a template molecule, thus highly selective to the specific contaminant. MIPs could specifically recognize and adsorb dioxin and related compounds, at least in the presence of some organic interferents; thus, they are applicable to environmental samples. For instance, water has been used for molecularly imprinted polymers specific for dioxins and chlorinated pesticides [36,37,38]. Functionalized nanoparticles involve adding chemical groups or biomolecules to the surface of nanomaterials to improve selectivity, stability, and dispersibility. Functional groups such as carboxyl, hydroxyl, and amine groups can enhance the interaction between nanomaterials and POP molecules, enabling better adsorption or catalytic degradation. Some functionalized nanomaterials are also designed to operate under specific pH or redox conditions, optimizing their activity in various environmental contexts [36].

### 3.4. Hybrid and Composite Nanomaterials

Hybrid and composite nanomaterials incorporate at least two of the following key components: carbon materials, metals, metal oxides, and polymers, presenting cooperative effects in POP degradation. These materials are designed to incorporate adsorption properties with photocatalysis and/or redox properties on the same platform to quicken degradation efficiency and the rate of the pollutants being removed by degradation [39,40]. For example, a nanocomposite of GO and TiO_2_ will adsorb the pollutants, and UV-active titanium dioxide will let the pollutants be degraded; GO can be separated by magnetic force after being saturated with pollutants. All these multifunctional materials overcome the drawbacks of single-function materials and are particularly desirable for treating wastewater of high complexity due to the diversity of classes of pollutants. In addition to the above, prior studies have investigated plasmonic nanocomposites, incorporating noble metals, i.e., silver or gold, with semiconductors to achieve extended light absorption in the visible range and enhance the efficiency of photocatalysis. These state-of-the-art materials are capable of solar-driven POPs degradation with the possibility of creating energy-efficient treatment processes for low-resource or remote settings [41,42].

## 4. Mechanisms of POP Removal Using Nanomaterials

POPs are highly toxic and exhibit chemical stability, enabling them to resist traditional degradation processes; thus, they pose a significant threat to both the environment and human life. Nanotechnology is a new technological approach to removing POPs, where each method targets specific physicochemical properties of nanomaterials. The following shall discuss the basic underlying mechanisms via which nanomaterials undertake the removal or degradation of POPs from aquatic environments, including adsorption, photocatalysis, redox transformations, magnetic separations, and hybrid strategies [1,43].

### 4.1. Adsorption Mechanisms

Adsorption is one of the primary mechanisms through which nanomaterials remove POPs from water. Nanomaterials like GO, CNTs, and activated carbon-based composites possess extremely high surface area and well-developed porosity, providing abundant active sites for pollutant interaction. The capacity is further improved by the existence of hydroxyl, carboxyl, or amine groups that can interact with POPs participating in hydrogen bonding, π–π stacking, van der Waals forces, or electrostatic attraction, respectively [15,31]. It augments selectivity and affinity for distinct POPs. For instance, aminated GO was more effective for the adsorption of chlorinated phenols as it provided stronger sites for the electrostatic interaction and hydrogen bonding raised by the introduction of the amino groups [36]. Several environmental and operational parameters that play a key role in adsorption performance are as follows: pH, since it affects the ionization of POP molecules and the surface charge of the adsorbent, thereby the strength and nature of interactions; temperature, which allows adsorption to be either enhanced or not depending on if it is endothermic or exothermic; and ionic strength which changes competitive adsorption dynamics, where a high concentration of competing ions may reduce the adsorption efficiency by occupying the active sites or destabilizing the adsorbate-adsorbent interaction [44]. Factors affecting the adsorption capacity of POPs are listed in Table 1.

### 4.2. Photocatalytic Degradation

The photocatalysis is conducted by activating semiconductor nanomaterials (e.g., TiO_2_, ZnO, Fe_2_O_3_, FeTiO_3_, Al_2_O_3_, and Cu_2_O) under UV or visible light [51]. On absorption of this light, electron-hole pairs are created. These charge carriers can then initiate redox reactions, resulting in the degradation of POPs. The process will depend on the semiconductors’ gap energy, light availability, and ability to separate and transfer charge carriers to the pollutant [18,34,52]. Photocatalytic degradation is primarily based on ROS, which means hydroxyl radicals (•OH), superoxide anions (O_2_^−^•), and singlet oxygen (^1^O_2_). These ROS will attack aromatic rings and halogenated groups in POPs, so dehalogenation and ring-opening can eventually lead to mineralization into CO_2_ and H_2_O. Photocatalysis does not provide a selective and strong decomposition approach for many kinds of POPs. However, its good results may be set by bad light going into muddy water and joining electron-hole pairs again, which can result in less ROS creation. To address this problem, strategies such as heterojunction engineering, such as coupling TiO_2_ with g-C_3_N_4_, metal, or nonmetal doping, such as nitrogen and silver, and core–shell structures are often used. These modifications enhance charge separation by generating an internal electric field, broaden the light absorption range, and reduce the probability of premature recombination of electron-hole pairs. This can improve the photocatalytic efficiency under practical environmental conditions [52,53].

### 4.3. Reductive Dechlorination and Redox Reactions

Reductive dechlorination is a mechanism via which the chlorine atoms of the POPs are replaced by hydrogen atoms using electron transfer. nZVI has been very frequently used as a reducing agent for this purpose. Its high surface area and electron-donating ability serve in breaking down compounds such as PCBs, dioxins, and chlorinated pesticides. Bimetallic nanoparticles like Fe/Pd or Fe/Ni accelerate the process by coupling nZVI with noble metals that catalyze the hydrogenation step [54,55]. Thus, it significantly increases the dechlorination rate and the number of dechlorinated compounds that can be treated. Electron transfer plays a central role in redox reactions involving POPs. The electrons donated by nZVI or other reducing agents attack electrophilic carbon–halogen bonds, leading to their cleavage and structural rearrangements, which occur mechanically. For halogenated POPs, detoxification through dechlorination also enhances biodegradability; reductive techniques find acutely useful applications in anoxic or reducing environments such as groundwater or deep sediments [35,56].

### 4.4. Magnetic Separation and Recovery

Magnetic nanomaterials such as magnetite (Fe_3_O_4_) and maghemite (γ-Fe_2_O_1_) are typically incorporated in the adsorption-based removal approaches for easy separation of the adsorbents or catalysts from the aqueous effluent. These materials can be functionalized with other adsorptive or catalytic components, such as hydroxyapatite, GO, or TiO_2_, creating multifunctional systems [32,57,58]. Once the pollutant is captured or destroyed, an external magnetic field can quickly retrieve the magnetic nanocomposites, thus minimizing material loss and secondary pollution. What gives magnetic nanomaterials an upper hand over others is reusability. After desorption or regeneration (through washing, heating, or UV treatment), these nanomaterials are applicable for several treatment cycles reused under the same conditions, which cuts operational costs and makes water treatment processes more sustainable [59,60,61]. Zhuo et al. [62] reported that the reusability of 83.5% methylene blue was recovered with adsorption capacity over five cycles of sorption/desorption by using magnetic carboxymethyl cellulose-based hydrogel nanosorbents. Siddiqa et al. [63] also found that the magnetite–maghemite nanocomposites adsorb 70% of Pb(II) and 57% of Cd(II) in five successive adsorption cycles. Only a 10% reduction in the removal efficiency of methylene blue was obtained after magnetite nanoparticles were reused for ten cycles of advanced oxidation [64]. The only downside is that performance tends to drop gradually after multiple usages owing to surface fouling, active component loss, and structural degradation; thus, periodical replacement and regeneration become necessary [32,65].

### 4.5. Hybrid and Synergistic Removal Mechanisms

**Figure 2:**
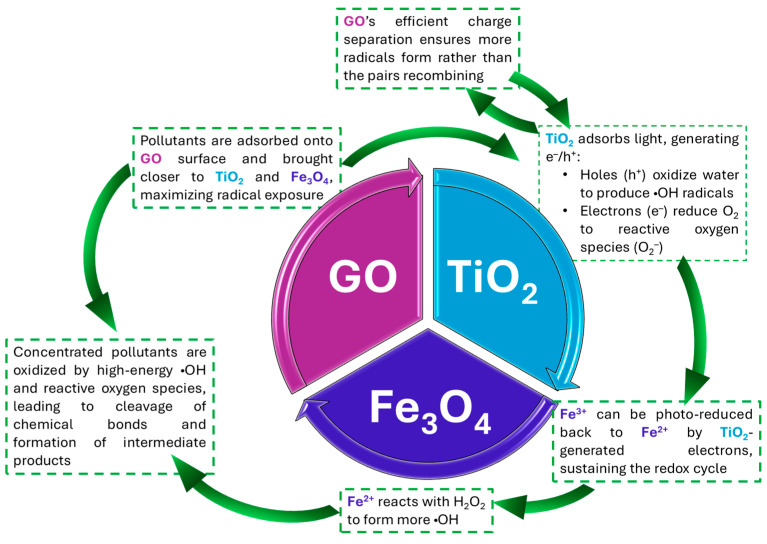
Synergystic mechanisms of POPs removal in GO–TiO_2_–Fe_3_O_4_ hybrid systems.

The use of a single nanomaterial platform that unifies adsorption, photocatalysis, and redox reactions can mark a paradigm shift in the technologies related to the remediation of POPs. This implies a synergistic effect beyond the mere sum of individual mechanisms [59]. An interesting possibility is represented by hybrid systems formed by GO–TiO_2_–Fe_3_O_4_ [66,67] (Figure 2). In these composites, graphene oxide serves as a high surface area adsorbent that brings pollutants closer to photocatalytic active sites, in this case, TiO_2_, which utilizes light energy for the generation of ROS capable of initiating oxidative degradation, and magnetic Fe_3_O_4_, which makes it possible to easily recover the composite material after treatment. The domains’ functions are placed in proximity to enhance reaction kinetics by reducing diffusion distances and allowing continuous degradation during adsorption, which minimizes secondary emissions and regeneration requirements. These composites can be engineered for specific contaminants and environmental conditions by adjusting their physicochemical properties, such as particle size, surface charge, and functional group density [67]. The unified system allows for treatment under broader pH ranges and variable pollutant loads and in the presence of natural organic matter, conditions under which typical single-mechanism approaches would often fail. This multifunctionality makes hybrid nanomaterials well-suited for treating complex, real-world wastewater matrices, as demonstrated by successful pilot-scale applications directed toward persistent pesticides, PCBs, and PAHs in agricultural and industrial settings [65,68].

**Figure 3:**
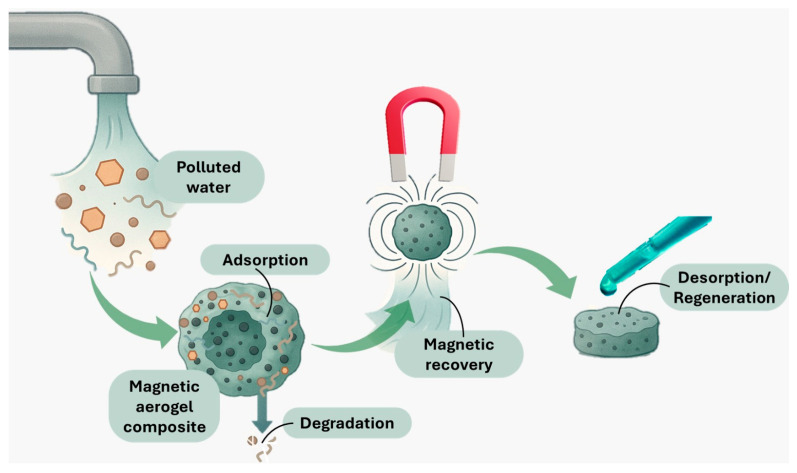
Schematic representation of the working principles of magnetic aerogel composites.

Interesting possibilities can also be envisaged by combining the retrievability of magnetic nanoparticles with the excellent surface area of aerogel-based materials (Figure 3). Aerogels consisting of silica-, polymer-, and carbon-based matrices have gathered increasing interest for applications in water remediation, especially due to their favorable porosity, versatility, tunability in terms of hydrophobicity/hydrophilicity, and recyclability (via contaminant desorption) [59,69,70,71]. Introducing the magnetic component to pristine aerogels was reported to improve their performance while also allowing easy separation from water samples, adsorbent regeneration, and further reuse [69]. Such magnetic aerogel composites have already been explored and have demonstrated their success for various organic pollutants, including dyes [72,73], organic solvents and oils [74,75], and pesticides [76], holding great promise for further investigations in mitigating POP contamination.

To more clearly distinguish between all the above-presented decontamination mechanisms, Table 2 offers an at-a-glance perspective on the topic.

## 5. Challenges and Limitations of Nanomaterial-Based POP Decontamination

While nanomaterials offer transformative potential for removing POPs from contaminated water, several critical challenges limit their widespread adoption and long-term application. These challenges range from technical and environmental constraints to economic and regulatory issues. Understanding and addressing these limitations is essential for developing safe, efficient, and sustainable nanotechnology-based water treatment solutions [10,44].

### 5.1. Nanomaterial Stability and Reusability

A major limitation to the application of nanomaterials in POP degradation is keeping their stability, effectiveness, and integrity over several cycles [81]. Many nanomaterials topically used for adsorption or photocatalysis tend to degrade or lose activity after several exposures to contaminants and environmental conditions. Factors that can reduce active surface area, block active sites, or change surface chemistry, such as photo corrosion (for TiO_2_), fouling, surface oxidation, and agglomeration, will all lower performance [82,83]. In addition, during use, the dissolution or piecemeal breaking up of nanomaterials may take place, leading to material loss in terms of both cost-effectiveness and environmental safety. For instance, passivated aggregation problems with nZVI prevent their reactivity from diminishing over time when employed for reductive dechlorination. More stable forms of nanostructures would have to be developed along with efficient recovery systems (like magnetic separation) designed to improve reuse and sustainability [35,38,79].

### 5.2. Formation of Toxic Byproducts

Another major issue is the development of toxic intermediates during the degradation of persistent organic pollutants [84]. While nanomaterials may efficiently promote the degradation of complex pollutants, they do not guarantee complete mineralization. In most cases, dangerous byproducts more toxic than the original compounds are formed instead of the full conversion into benign end-products such as CO_2_ and H_2_O. For example, photocatalytic degradation can generate hydroxylated intermediates or semi-oxidized organics, which will be persistent in the environment [42]. Li et al. [85] examined aromatic pollutant degradation via TiO_2_ photocatalysis and found varying distribution of monohydroxylated isomers (e.g., o-, m-, and p-hydroxy-benzene derivatives). These concluded that incomplete oxidation leaves persistent OH-intermediates. Sobczynski et al. [86] investigated phenol degradation using TiO_2,_ observing primary intermediates such as catechol, hydroquinone, and benzoquinone, along with short-chain oxidation products, highlighting the risks associated with persistent semi-oxidized organic compounds. Metal nanoparticles, on their part, may also result in reductive dechlorination to the accumulation of resistant fragments chlorinated to further degradation [78]. Iron nitride and nZVI studies on TCE degradation showed that aged nZVI achieved only 75% Cl- recovery, indicating that 25% of chlorine remained in residual chlorinated intermediates [87]. TCE degradation by nano-iron may stall at DCE or vinyl chloride (VC), both of which are more toxic and persistent than TCE itself [88]. The hydroxylated intermediates possess increased cytotoxicity, mutagenicity, and endocrine-disrupting potential [89], whereas the metal nanoparticles can induce oxidative stress, inflammation, and cytotoxicity in various organisms [90]. Without appropriate toxicity profiling, secondary pollutant formation may be overlooked. Thus, detailed mechanistic studies coupled with ecotoxicological assessments are necessary for tracking degradation byproducts and their impacts as a basis for designing safer nanomaterials and treatment protocols [91].

### 5.3. Environmental Fate and Potential Toxicity of Nanomaterials

Though nanomaterials help eliminate pollutants, they have an equally adverse environmental impact. Once these particles are either deliberately or accidentally introduced into any aquatic system, their behavior with living organisms and ecosystems is completely unpredictable. They possess very small dimensions and high reactivity, making them bioavailable to most aquatic species; however, they are likely to induce toxic effects related to oxidative stress, membrane damage, or disruption of cellular processes [92,93]. Nanoparticles, such as silver nanoparticles (AgNPs) and zinc oxide nanoparticles (ZnO NPs), have been shown to accumulate in fish, algae, and invertebrates, raising concerns about trophic transfer and long-term ecological impacts. Bioaccumulation pathways include direct uptake through gills or cell membranes and indirect uptake through ingestion of contaminated prey, which can lead to biomagnification throughout the food web. Species sensitivity distribution (SSD) models are increasingly being used to predict the ecological risks of nanomaterials. The models estimate the concentration at which a specific percentage of species will be adversely affected [93,94,95]. Ecotoxicological regulation, including standardized testing based on OECD guidelines, is essential for assessing environmental hazards. However, the current testing protocols often lack specificity for nanoscale properties, indicating the need for improved or new testing methods. Therefore, comprehensive fate and transport studies, life cycle assessments, and regulatory frameworks for nanomaterials are essential to ensure their safe and sustainable use in environmental remediation [93,96].

### 5.4. Scalability and Economic Feasibility

Despite the promising performance of nanomaterials at the laboratory scale, scaling them up for real-world applications poses major logistical and financial challenges [97]. The synthesis of high-quality nanomaterials usually has precursors that are not cheap, with complicated setups and processes that consume a lot of energy; all these factors will add to the cost of production and hamper its economic feasibility. In addition, utilizing nanomaterials in at least municipal or industrial treatment water volumes demands assured quality control as well as recovery methods and systems integrated into the existing infrastructures for treatments. Such requirements will increase operational costs on the one hand and limit commercial interest on the other, especially in resource-constrained settings [98]. Even though alternative synthesis routes using waste biomass, green chemistry techniques, or scalable reactor designs have been considered to alleviate some of these problems, nanotechnology transfer from the lab to the shop floor has been overworked by underworked hands. Cost-efficient, scalable, and environmentally safe nanotechnologies can only be developed through collaboration between academia, industry, and government [1,10,39].

### 5.5. Regulatory and Safety Concerns

A major barrier to the large-scale application of nanomaterials in environmental remediation, particularly in water treatment, is the lack of clear and standardized regulations. Currently, there are no comprehensive, globally agreed-upon guidelines for the production, use, and disposal of engineered nanomaterials in the environmental sector [99,100]. This regulatory gap creates uncertainty for stakeholders, slows the commercialization of nanotechnology, and undermines the public acceptance of nanotechnology [101,102]. Another difficulty in risk assessment stems from the diversity of properties and behaviors of nanomaterials in different environmental matrices. Traditional toxicity tests, which are typically developed for bulk chemicals, are not always applicable to assess the unique risks posed by nanoparticles. In addition, reliably monitoring the concentration, transformation, and persistence of nanoparticles over time remains a challenge [99,101,103,104]. Some progress has been made at the national and regional levels. For example, the EU’s Registration, Evaluation, Authorization and Restriction of Chemicals (REACH) framework has introduced specific provisions for nanomaterials, requiring detailed physicochemical characterization, exposure scenarios, and risk assessments. The U.S. Environmental Protection Agency (EPA) has begun reviewing new nanomaterial applications under the Toxic Substances Control Act (TSCA), but specific protocols for nanomaterials have not yet been developed in several areas. Both frameworks emphasize the need for sound risk assessment tools for nanomaterials, including life cycle assessment and post-use environmental monitoring. To address these challenges, international collaboration is essential to establish uniform standards for nanomaterial characterization, toxicity testing, and environmental monitoring. Regulators should work closely with academia and industry to define safe exposure limits, enforce labeling requirements, and develop clear guidelines for waste treatment and end-of-life management [104,105,106].

For better clarity, the main challenges and limitations of nanomaterials in POP decontamination are summarized in Table 3.

## 6. Case Studies and Real-World Applications

Nanomaterials have gained significant traction in environmental remediation owing to their unique properties and multifunctional capabilities. Several case studies across countries such as Egypt, Azerbaijan, and Italy have demonstrated their efficiency in removing POPs from water, positioning nanotechnology as a promising alternative to conventional remediation methods [10,108]. For example, in Egypt, a pilot-scale study utilizing a graphene oxide–titanium dioxide–magnetite (GO–TiO_2_–Fe_3_O_4_) composite treated agricultural runoff contaminated with chlorinated pesticides. Under natural sunlight, the composite achieved over 90% degradation of chlorinated pesticides from agricultural runoff under natural sunlight within 4–6 h, outperforming conventional activated carbon. The study also reported efficient magnetic recovery and reusability over three cycles with minimal efficiency loss [109]. In Azerbaijan and Italy, the use of Fe/Ni bimetallic nanoparticles supported on activated carbon resulted in PCB concentrations dropping from initial levels of 120–150 µg/L to below 0.5 µg/L within one week—well under the local regulatory thresholds [54,56].

Comparative analyses between these nanomaterial technologies and conventional remediation approaches reveal several advantages. Traditional methods such as coagulation–flocculation, sedimentation, and biological treatment often struggle with the recalcitrant nature of POPs due to their low water solubility and high chemical stability [110]. In contrast, nanomaterial-based treatments harness high surface areas and tunable surface functionalities to facilitate effective adsorption and degradation. For instance, while activated carbon might sequester a fraction of the POP load, its limited catalytic capability often necessitates additional oxidation steps to achieve complete degradation. Nanomaterials, particularly hybrid systems that integrate adsorption, photocatalysis, and redox reactions, can combine these functions in a single step, offering faster treatment times and higher overall efficiency [111].

However, the transition from laboratory to field-scale implementations is beset with a different set of challenges. A major challenge is the consistency and cost-effectiveness of scaling nanomaterials production from grams to tons for application in real-world situations [112,113]. Field implementations in countries like Saudi Arabia have demonstrated that, although very high removal efficiencies can often be achieved in the laboratory under controlled conditions, the heterogeneous and dynamic nature of contaminated sites can significantly reduce the effectiveness of such systems [114,115]. For instance, a field-scale demonstration of nZVI-based groundwater remediation reported a decline in dechlorination efficiency, which was attributed to partial agglomeration caused by fluctuating aquifer conditions, such as changes in pH, ionic strength, and groundwater flow dynamics [116]. This observation underscores the importance of developing robust synthesis and stabilization strategies that can preserve the reactivity and mobility of nanomaterials under heterogeneous and unpredictable subsurface environments. Current possibilities include coating nZVI with silica, biodegradable polymers, or surfactant-based dispersants to reduce agglomeration and oxidative passivation [117,118,119,120]; however, these approaches remain insufficiently validated at larger scales and are not yet widely implemented in field conditions.

Field-scale studies have also identified some logistical and environmental challenges to the deployment of nanomaterials in situ. Comprehensive risk assessments and long-term monitoring are called for due to potential nanoparticle leaching, interactions with native biota, and unforeseen changes in water chemistry. Lessons from early implementations underscore the need for integrating engineered nanomaterials within the existing treatment frameworks to minimize these risks [120,121]. In addition, several projects have indicated that rigorous site characterization, including hydrogeological assessment and pre-treatment contaminant mapping, is critical for effective remediation strategy design using nanotechnology [122,123].

A comparative analysis of the varied nanomaterial strategies reveals the different advantages and limitations inherent in each approach. Adsorption-based strategies involving carbonaceous materials such as graphene oxide and carbon nanotubes are efficient in capturing hydrophobic POPs due to π–π interaction and van der Waals force but normally do not have intrinsic degradative capabilities, leading to subsequent treatment steps [15,31]. The photocatalytic procedures based on semiconductors like TiO_2_ and ZnO will solve this problem since they actively decompose POPs under the irradiation of light. However, it poses a challenge due to charge-carrier recombination and requires UV light, which may limit its efficiency under natural sunlight in some regions [42,82,109]. The reductive dechlorination predominantly catalyzed by nZVI and its bimetallic variants is very efficient for halogenated POPs but somewhat susceptible to the effects of passivation as well as their agglomeration over time, which decreases their reactive surface area [35,38,79].

Hybrid and composite nanomaterials, which amalgamate multiple functional mechanisms into a single system, have emerged as particularly promising due to their ability to combine the strengths and mitigate the weaknesses of individual approaches [39,40]. For instance, a composite that integrates the adsorption capacity of GO, the photocatalytic properties of TiO_2_, and the magnetic recoverability of Fe_3_O_4_ not only enhances overall removal efficiency but also facilitates easy separation and reuse [15,31,42]. Case studies from some countries demonstrate that such multifunctional systems can achieve rapid and comprehensive POP removal, even in complex water matrices, thereby offering a viable pathway to scalable, sustainable water treatment solutions [10].

## 7. Future Directions and Research Gaps

The field of nanotechnology has demonstrated significant promise in addressing the challenge of removing POPs from water systems. However, as the field matures, future research must address several gaps and areas for advancement to ensure that nanomaterial-based approaches are not only effective but also safe, scalable, and environmentally sustainable [10,18,26]. Key areas of future exploration include the design of improved nanomaterials, adopting green synthesis techniques, monitoring technology advances, integrating with conventional systems, and comprehensive environmental impact assessments [32,52,79].

A central priority for future research lies in designing advanced nanomaterials that are both multifunctional and highly selective for various classes of POPs. Current nanomaterials often exhibit strong performance in specific applications such as adsorption or photocatalysis [18,34,68], but may lack efficiency across a broader range of contaminants or operating conditions. There is a growing need for materials that combine multiple functional mechanisms (adsorption, catalysis, and redox reactivity) to enable synergistic performance [39,65]. For example, developing nanocomposites that simultaneously adsorb and degrade different POPs can reduce treatment time and complexity. Enhancing selectivity—through surface modification or molecular imprinting—can also improve targeting specific pollutants in complex water matrices. Alongside functionality, recyclability and sustainability must be improved. Many existing nanomaterials suffer from limited reusability, agglomeration, or activity loss over repeated cycles. Future materials should be engineered for durability and designed with easy recovery mechanisms to reduce costs and environmental burdens [10,65,82,107].

Parallel to functional improvement, developing green and biodegradable nanomaterials is imperative. As the deployment of engineered nanomaterials increases, concerns about their potential environmental and human health impacts have also grown. Conventional synthesis methods often involve toxic solvents, high-energy inputs, or hazardous reagents. Transitioning to eco-friendly synthesis routes, such as plant extract-mediated synthesis, sol–gel methods under ambient conditions, or microbial-assisted fabrication, can reduce these environmental footprints [10,107,124]. Moreover, using bio-inspired or biodegradable nanomaterials, such as cellulose-based nanostructures, protein-coated nanoparticles, or chitosan composites, aligns with the principles of green chemistry and reduces the risk of secondary pollution. Such materials may offer sufficient performance while decomposing safely in the environment, making them more suitable for large-scale deployment in natural water systems [10,15,29].

Another pressing need lies in the advancement of analytical and monitoring technologies to track POPs and assess the performance of nanomaterials in real time. Traditional detection techniques, such as gas chromatography–mass spectrometry (GC-MS), are highly accurate but require expensive instrumentation and are not suitable for field conditions. Future efforts must focus on developing portable, high-sensitivity detection tools that can operate in situ [125,126]. Techniques such as surface-enhanced Raman spectroscopy (SERS), electrochemical sensors, and fluorescence-based assays are gaining traction due to their potential for rapid and sensitive detection. Additionally, real-time monitoring systems that can continuously assess water quality during and after treatment are essential for ensuring the efficacy and safety of remediation processes. These technologies can also help optimize treatment parameters on the fly and reduce the risk of incomplete pollutant removal [126,127,128].

Equally important is the integration of nanotechnology with the existing water treatment infrastructures. Rather than functioning as standalone systems, nanomaterials can be strategically embedded into current treatment processes to enhance their overall efficiency. For instance, incorporating nanomaterials into membrane filtration systems can improve anti-fouling properties and pollutant rejection rates [9,15]. Similarly, electrochemical treatments augmented with catalytic nanomaterials may offer enhanced oxidative or reductive transformation of POPs [110,126]. Biological treatment approaches (e.g., constructed wetlands or biofilters) can be combined with nanomaterials to exploit microbial and material-based degradation pathways [53,84]. Furthermore, smart nanomaterials with self-regenerating, self-cleaning, or stimulus-responsive properties hold the potential for autonomous operation in dynamic environmental settings. For example, nanomaterials that regenerate under solar light or respond to changes in pH or temperature can reduce maintenance needs and extend operational lifetimes [1,8,10].

Despite the promising capabilities of nanomaterials, their long-term environmental impact and potential risks remain inadequately understood. Many nanomaterials, especially metal-based and synthetic polymers, can persist in the environment or exhibit unintended toxicity toward aquatic organisms and ecosystems. Therefore, comprehensive risk assessment frameworks must be established to evaluate their fate, transport, transformation, and ecotoxicity in diverse environmental contexts [35,39]. Research should address how nanomaterials interact with natural organic matter, how they behave in sediments, and whether they bioaccumulate in food chains. Additionally, regulatory frameworks for deploying nanomaterials in water treatment must be clarified and standardized across jurisdictions. Guidelines for allowable concentrations, disposal methods, labeling, and monitoring responsibilities will be essential to support safe and responsible implementation [97,107].

**Figure 4:**
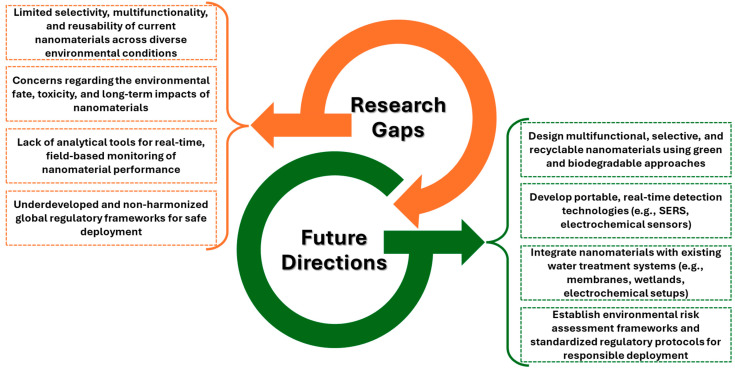
Key research gaps and future study directions in the application of nanomaterials.

For an at-a-glance perspective, the identified research gaps and future study directions have been summarized in Figure 4.

## 8. Conclusions

Nanotechnology has emerged as a powerful tool in the fight against POPs in water systems. Through extensive research and growing real-world applications, nanomaterials have demonstrated exceptional effectiveness in POP removal, primarily due to their high surface area, tunable surface chemistry, and multifunctional properties. From adsorption and photocatalytic degradation to reductive dechlorination and hybrid removal systems, a wide range of nanomaterials—including carbon-based, metal oxide, and functionalized composites—have consistently outperformed traditional treatment methods in both efficiency and versatility. Case studies further validate these findings, illustrating successful pilot- and field-scale applications across diverse environmental contexts.

Despite these achievements, challenges remain. Ongoing concerns include scaling up nanomaterial production while maintaining consistency and cost-effectiveness, mitigating potential ecotoxicological impacts, and ensuring long-term material stability. Additionally, transitioning from laboratory innovation to full-scale deployment requires overcoming technical, regulatory, and environmental hurdles. Lessons learned from early field implementations underscore the importance of integrating nanomaterials into the existing infrastructure, designing adaptable systems to local water chemistry, and prioritizing safe material recovery and reuse.

To address these gaps, future research should focus on (i) developing next-generation nanomaterials with improved selectivity, reusability, and environmental compatibility; (ii) scaling up green synthesis processes using plant-based or microbial approaches; (iii) integrating machine learning techniques to optimize nanomaterial design and predict degradation outcomes; and (iv) conducting long-term ecotoxicity and life cycle impact assessments under realistic environmental scenarios. In addition, regulatory developments must keep pace with innovation. A coordinated roadmap involving academia, industry, and policymakers is needed to establish clear standards for nanomaterial classification, risk assessment, environmental monitoring, and end-of-life management. Through interdisciplinary collaboration and forward-looking policies, nanotechnology has the potential to revolutionize water treatment and provide safe, efficient, and sustainable solutions for POPs removal in both developed and developing countries.

## Figures and Tables

**Table 1:** Factors affecting the adsorption capacity of POPs.

Parameter	Adsorption Capacity of POPs (PCBs/OCPs)	
	GO [45,46,47]	CNTs [48,49,50]
pH	GO contains abundant oxygen-containing functional groups (carbonyl, hydroxyl, and epoxy), which ionize depending on pH, affecting surface charge and hydrophilicity. Adsorption capacity for PCBs and OCPs often decreases at high pH due to increased negative charges causing repulsion or reduced hydrophobic interaction. Adsorption capacity decreases by up to 30–50% when pH shifts from pH 4 to pH 9.	CNTs are generally hydrophobic with limited surface functional groups, so adsorption of hydrophobic pollutants like PCBs and OCPs is less sensitive to pH changes. Slight decreases in adsorption capacity at extreme pH due to surface charge alterations and possible aggregation. Adsorption capacity changes <15% across pH 3–9 for PCBs/OCPs.
Temperature	Adsorption of PCBs and OCPs may be more sensitive to temperature due to involvement of hydrogen bonding and electrostatic interactions. Adsorption capacity often decreases with increasing temperature, but sometimes shows slight increases if diffusion is rate-limiting. Adsorption capacity change varies widely ±10–20% via improved kinetics and thermodynamic favorability.	Adsorption is generally exothermic; increasing temperature typically decreases adsorption capacity. Adsorption capacity may decline by 10–30% when the temperature increases from 20 to 40 °C.
Ionic strength	Higher ionic strength screens electrostatic repulsion between negatively charged GO species, potentially increasing adsorption capacity. At very high ionic strengths, the aggregation of GO may reduce the available surface area. Adsorption may increase up to 30% at moderate ionic strength (0.05–0.1 M), but decline beyond this due to aggregation effects.	Increasing ionic strength (e.g., NaCl concentration) can enhance adsorption slightly via salting-out effects that reduce pollutant solubility, promoting partitioning onto CNTs. Electrostatic screening is minimal due to CNTs’ low surface charge. Adsorption capacity may increase by ~10–20% with ionic strength from 0 to 0.1 M NaCl.

Abbreviations: POP—persistent organic pollutant; PCB—polychlorinated biphenyls; OCP—organochlorine pesticides; GO—graphene oxide; CNT—carbon nanotubes.

**Table 2:** Mechanisms employed by nanomaterials for the removal of POPs.

Mechanism	Description	Key Features	Comparative Efficiency	Refs.
Adsorption	Physical or chemical capture of POPs on nanomaterial surfaces via surface interactions. Nanomaterials such as GO, CNTs, and activated carbon provide abundant active sites and tunable functionalities.	High surface area; π–π stacking, hydrogen bonding, and electrostatic attraction; enhanced selectivity via functional groups; performance influenced by pH, temperature, and ionic strength.	~60–95% depending on material type, surface area, and conditions	[15,31,36,44]
Photocatalytic Degradation	Semiconductor nanomaterials (e.g., TiO_2_ and ZnO) absorb UV or visible light to generate electron-hole pairs, which initiate redox reactions and produce ROS that degrade POPs.	ROS generation (•OH, O_2_^−^•, and ^1^O_2_); capable of mineralization to CO_2_ and H_2_O; effective for a broad range of POPs; limited by light penetration and electron-hole recombination.	~70–99% under optimized light intensity and catalyst loading	[18,34,52,53,77]
Reductive Dechlorination/Redox Reactions	Electron transfer reactions, particularly from nZVI or bimetallic nanoparticles, reduce halogenated POPs by replacing chlorine with hydrogen atoms, breaking C–Cl bonds.	Effective for halogenated POPs (PCBs and dioxins); rapid dechlorination via nZVI or Fe/Pd and Fe/Ni systems; enhances biodegradability; applicable in anaerobic or reducing environments.	~65–98% depending on POP type and nanoparticle composition	[35,56,78,79]
Magnetic Separation and Recovery	Using magnetic nanomaterials (Fe_3_O_4_ and γ-Fe_2_O_3_) allows rapid separation and recovery of adsorbents or catalysts from treated water via external magnetic fields.	Easy separation post-treatment; integration with catalytic/adsorptive materials (GO and TiO_2_); reusability after regeneration; reduces material loss and operational costs; performance can decline with repeated cycles.	Removal remains ~70–90% initially, may decline after reuse cycles	[32,57,65]
Hybrid and Synergistic Mechanisms	Multifunctional nanocomposites (GO–TiO_2_–Fe_3_O_4_) integrate multiple mechanisms (adsorption, photocatalysis, redox) in one platform for synergistic pollutant removal.	Combines advantages of individual mechanisms; higher degradation efficiency; rapid kinetics; magnetic separability; tailored for broad pH, pollutant types, and complex matrices; supports continuous degradation with reduced regeneration frequency; scalable and robust for field applications.	Up to 99% removal; enhanced synergy under combined treatment	[15,31,53,65,68,80]

**Table 3:** Key challenges limiting the widespread application of nanomaterials in POP decontamination.

Challenge	Description	Refs.
Nanomaterial Stability and Reusability	Structural degradation, surface fouling, aggregation, and photo corrosion reduce nanomaterial performance over time. nZVI is prone to passivation and agglomeration. Enhancing material robustness and recovery methods is essential for long-term use.	[35,38,79,81,82,83]
Formation of Toxic Byproducts	Incomplete mineralization can lead to hazardous degradation intermediates, including hydroxylated or chlorinated byproducts. Photocatalysis and reductive reactions often leave residual toxicity. Comprehensive degradation pathway studies and ecotoxicological assessments are needed.	[42,78,84,91]
Environmental Fate and Nanotoxicity	Nanoparticles may accumulate in aquatic organisms, alter microbial communities, and pose risks to ecosystems and human health. Understanding their transport, transformation, and long-term toxicity is critical. Green synthesis and lifecycle assessments can mitigate environmental impact.	[92,93,96]
Scalability and Economic Feasibility	High production costs, complex synthesis methods, and difficulty integrating into the existing infrastructure limit scalability. Green and low-cost synthesis approaches, as well as pilot-scale demonstrations, are needed to ensure economic viability.	[1,10,39,97,98]
Regulatory and Safety Concerns	Lack of standardized regulations, monitoring tools, and toxicity testing protocols hinders responsible deployment. The current risk assessments often overlook nanoparticle-specific behaviors. Harmonized international standards are essential for commercialization and public acceptance.	[97,105,107]

## Data Availability

No new data were created or analyzed in this study.
